# Prevalence of depression and anxiety among newly diagnosed cancer patients: a single centre experience in the Middle East

**DOI:** 10.3332/ecancer.2024.1690

**Published:** 2024-04-04

**Authors:** Mona Ali Hassan, Ahmad EL Mahmoud, Suha Kalash, Tamara Kadi, Nour Bakhos, Reine Abou Zeidane, Ghid Amhaz, Maya Bizri, Hazem I Assi

**Affiliations:** 1 Department of Internal Medicine, Division of Hematology-Oncology, Naef K. Basile Cancer Institute, American University of Beirut Medical Center, Beirut, Lebanon; 2 Department of Internal Medicine, American University of Beirut Medical Center, Beirut, Lebanon; 3 Department of Psychiatry, American University of Beirut Medical Center, Beirut, Lebanon

**Keywords:** anxiety, cancer, depression, Lebanon, mood disorders, neoplasms, oncology, patient health questionnaire, prevalence, psycho-oncology

## Abstract

Failure to identify and treat depression and anxiety affecting 10% of patients with cancer, increases the disease burden. This study aimed to assess the psychological well-being of newly diagnosed patients in a tertiary healthcare centre in Lebanon. In this cross-sectional study, data were collected for 187 adult patients, from medical records and interviews using standardised questionnaires (Personal health questionnaire-9 (PHQ-9) and generalised anxiety disorder-7). Karnofsky performance status was also assessed, and incidence was calculated using descriptive statistics, chi-square, and *T*-tests. The rates of moderate or severe anxiety, minimal anxiety, mild depression, moderate or severe depression, and suicidality are 14.9%, 35.6%, 40.7% 22.7% and 6.2%, respectively. Participants with a past history of seeking help from mental health services (OR: 3.978, CI: (1.680–9.415), *p* = 0.002), those developing cancer-related complications (OR: 3.039, CI: (1.187–7.777), *p* = 0.020), and those who had an Eastern Cooperative Oncology Group of ≥2 (OR: 5.306, CI: (1.582–17.797), *p* = 0.007) were independently associated with depression (diagnosed with PHQ-9) in multivariate logistic regression analysis. Patients with cancer exhibit higher evidence of depression and anxiety and should have a thorough psychiatric history and additional psychiatric care.

## Background

Psycho-oncology is a relatively new oncology subspecialty that makes a significant contribution to cancer care. The recent increase in the incidence of cancer and lifespan of patients with cancer has been attributed to the newly developed methods of diagnosis, treatment and increased screening [1].

Psychiatric illnesses such as depression and anxiety are common among patients with cancer and affect up to 25% and 18% of patients with cancer, respectively, regardless of the goal of oncological management, be it curative or palliative [2].

Lebanon has the highest incidence of cancer among Arab countries, and there has been a steady increase in the incidence of cancer cases from 2005 to 2016 [3]. According to the World Health Organisation, the total number of cancer cases in 2020 is approximately 11,589. The 5-year prevalence of cancer is estimated to be 421 per 100,000 people. The risk of developing cancer before the age of 75 years is 16.4% (compared to 20.4% globally). The top five most frequent cancers, excluding non-melanoma skin cancer, for both sexes are breast, lung, prostate, colorectal and bladder cancers [4].

Cancer diagnosis can cause high levels of stress, especially during the initial diagnosis and treatment phase; however, in the Middle East in general and in Lebanon, in particular, little is known about the prevalence of anxiety and depression among newly diagnosed patients with cancer. Despite the fact that early screening for depression and anxiety is recommended by several studies and guidelines [5], it is still not part of the standard of care in Lebanon. This study aimed to assess the prevalence of depression and anxiety and their risk factors among newly diagnosed cancer patients receiving treatment at the American University of Beirut Medical Center (AUBMC), a tertiary care centre that caters to up to 4,000 patients with cancer per year.

## Methods

This was a cross-sectional, descriptive survey that included patients with cancer actively receiving treatment at the AUBMC. Patients were interviewed over a 1-year period from January 2021 to 2022. Inclusion criteria were age above 18 years, cancer diagnosis within 6 months, and active treatment at AUBMC. Minors (<18 years of age) and those who received a bone marrow transplant were excluded because of more complicated treatment courses. For patients who agreed to participate, the study’s aim and objectives were explained, and information sheets were provided for further clarification about the study. After obtaining written consent from the patients, data regarding malignancy characteristics, treatment modalities, and patients’ performance status were collected from medical and electronic health records. Interviews were conducted using standardised questionnaires: the nine-item personal health questionnaire (PHQ-9) to assess depression and the seven-item generalised anxiety disorder-7 (GAD-7) questionnaire to assess anxiety. The PHQ-9 is a tool that can be used to screen for and assess for severity of depression. It is made up of nine questions that are graded from 0 to 3. The highest score is 27 and the lowest score is 0. PHQ-9 scores ≥10 had a sensitivity of 88% and a specificity of 88% for major depression. The cutoffs usually used are 5, 10, and 15 for mild, moderate, and severe depression [12]. The PHQ-9 also includes a question about the risk of suicidality and/or passive death wishes, which was also singled out for use in the data analysis. The GAD-7 is a similar tool that can be used for screening and assessing the severity of anxiety. A cut off score of 10 is considered to be reliable for GAD. Similar to the PH-q, scores of 5, 10 and 15 are used as cut offs for mild, moderate and severe anxiety. (The Eastern Cooperative Oncology Group (ECOG) score was assessed during the interviews [13]. The score is a scale that can be used to assess the level of activity of patients and the effects of the progression of the disease on their ability to perform activities of daily living. Grade 0 indicates normal activity levels, Grade 1 suggests some limitations in physically demanding tasks but can perform light work, Grade 2 indicates the ability for self-care but not work activities, Grade 3 signifies limited self-care ability with more than 50% of time spent in bed or chair, Grade 4 indicates complete inability for self-care and confinement to bed or chair, and Grade 5 represents death. We considered a score of 0–1 to be low while any score above 2 was considered high. The variables collected included age, gender, type of cancer (specifically whether it was hematologic or solid), intent of treatment (curative intent versus palliative intent), complications (measured by number of hospital admissions for complications), type of cancer treatment received, and psychiatric history, which included a history of mental illness (including major depressive disorder and GAD), seeking help from a mental health professional, and taking psychoactive medication (including antidepressants). Incidence and descriptive statistics were calculated, and the variables were compared using SPSS software using binary logistic regression to determine the risk factors that were significantly associated with depression, anxiety, and suicidality. A significant (*p* < 0.05) logistic regression output was observed.

## Results

Overall, 194 patients were interviewed, and their data were analysed. Characteristics investigated included age, gender, type of malignancy, disease status at diagnosis, ECOG PS, treatment intent, treatment modalities, history of psychiatric illness and hospitalisation number.

Participants had a mean age of 55.73 years (range, 18–90 years), with 56.12% females and 43.88% males. Solid malignancies were present in 80.90% of cases, with breast cancer (21.6%) being the most common, and haematological malignancies in 19.10% cases, with lymphoma (12.4%) being predominant. Thirty-seven percent had de novo metastatic cancer. Quality of life, assessed by ECOG performance status, indicated 55% had a score of 0. The majority (93%) received treatment for cure or control, primarily chemotherapy (88.1%). Surgical intervention was necessary in 31.4% of cases. Hospitalisation incidence varied: 56.2% had no admissions, 28.9% had one, 8.8% had two, and 6.1% had three or more. Prior to diagnosis, 16% had a history of mental health disorders, 17% sought professional mental health support, and 13.4% were taking psychiatric medication. Detailed baseline characteristics are in Table 1.

In our population, we screened for the risk of both anxiety (using the general anxiety disorder-7 questionnaire) and depression (using the patient health questionnaire–9). As shown in Table 2, 35.6% of our population reported minimal levels of anxiety (GAD 7 ≥5), and 14.9% reported moderate or severe levels of anxiety (estimated at a score of 10 or higher). The rate of mild depression (PHQ-9 ≥5) was 40.7%, while that of moderate or severe depression (PHQ-9 ≥10) was 22.7%. Suicidality was detected in 6.2% of patients, all of whom were immediately referred to psychiatric care. All of the collected characteristics were analysed further to determine which ones were associated with an increased rate of anxiety and depression in our patients.

The results of multivariate analysis presented in Table 3 show that a history of seeking help from mental health services prior to a cancer diagnosis was four times associated with the risk of developing depression (*p* = 0.002). Patients who developed cancer and cancer treatment-related complications were three times more likely to be at risk of developing depression (*p* = 0.02), whereas those who received radiotherapy were 2.5 times more likely to be at risk of developing depression (*p* = 0.026). Patients with an ECOG of ≥2 were five times more likely to be at risk of developing depression (*p* = 0.007).

It is very important to note that a history of seeking help from mental health services (Table 3) was associated with an 8.4-fold risk of developing suicidal thoughts or passive death wishes (*p* = 0.001).

## Discussion

Despite advancements in early detection and medical treatment, the diagnosis of cancer continues to be a source of great distress and anxiety for patients and their families, regardless of the prognosis of the disease and its stage [5]. The American Society of Clinical Oncology recommends that all patients with cancer be evaluated for symptoms of depression and anxiety at their initial visit, at appropriate intervals, and as clinically indicated, especially with changes in disease or treatment status (i.e., post-treatment, recurrence and progression) and transition to palliative and end-of-life care [5]. For example, according to Burgess *et al* [6] the occurrence of depression among breast cancer patients was 33% at the time of diagnosis, 15% a year after diagnosis and 45% after relapse [6].

Even before the start of treatment, the fear of disease progression, pain and side effects of cancer treatment can increase patients’ levels of anxiety and depression [7]. In a large meta-analysis of 24 studies with 4,007 individuals across seven countries, Mitchell *et al* [2] reported that the prevalence of anxiety disorders was 9.8%. The prevalence of all types of depression was 24.6% [2]. Similarly, according to Karam *et al* [8], the lifetime prevalence of anxiety disorders in Lebanon was 25.8%, mood disorders was 12.6%, impulse control disorders was 4.4% and that of substance-use disorders was 2.2%, which is similar to that of our cohort.

Our study identified a possible risk factor for the development of anxiety, depression and suicidality among newly diagnosed cancer patients: we found that the presence of a history of seeking mental health assistance was highly correlated with all three. Therefore, the psychological background of patients should be considered when stratifying patients at the time of initial screening prior to the start of therapy. These patients should be followed-up more closely and their mental health should be assessed more frequently than their contemporaries.

Our study also found that patients who received radiotherapy had a higher incidence of depression. This is pertinent because approximately 50% of all patients with cancer receive radiation therapy during their illness [9]. The RTOG 0841 trial published in 2017 investigated screening for depression among patients with cancer receiving radiotherapy; 16.5% of patients exceeded screening cut-offs for depressive symptoms and underwent further investigation [10]. Stiegelis *et al* [11] showed that 21%–54% of patients developed feelings of anxiety and 12%–31% developed symptoms of depression during radiation treatment; therefore, our findings are not inconsistent with the literature. This may be related to the physical side effects of radiotherapy, time commitment and frequent daily visits to the hospital for radiation therapy patients or the fact that these patients might not have recovered psychologically from previous lines of treatment (surgery or chemotherapy followed by radiation therapy).

Recurrent hospitalisation due to disease complications was also found to increase the rate of depression in patients. This seems logical because complications imply treatment delays, more time spent in the hospital, an increase in the cost of treatment, more pain and suffering, and an overall worse prognosis.

Interestingly, we found no association between chemotherapeutic treatments and the development of mood disorders.

This study has several strengths, including the use of standardised measures for the assessment of anxiety and depression and the clinically meaningful analysis arising from the Middle Eastern population, where public health awareness about mental illness is still lacking in comparison to other areas around the world.

One of the limitations of this study is that the data collection took place at the start of the financial crisis in Lebanon. At that time, antineoplastic medications were available and affordable. If the study were to be conducted at this time, the severe shortage of medications, the rise of the black market, and the financial burden on the patients would have to be taken into account. Nevertheless, the limitation of the tools used as PHQ-9 and GAD-7, even though they are most validated in Arabic, is that these tools consider somatic symptoms when they are being scored, so they could be confounded by the cancer symptoms themselves as well as by the chemotherapy side effects and radiation side effects, including chemotherapy brain fog. We speculate that this is reflected in the higher prevalence of depression and anxiety among the patients. An additional consideration in this study is the inclusion of patients with prior or existing mental health disorders, including conditions such as GAD and major depressive disorder. While this may introduce complexity and potentially confound results, we deemed it essential to incorporate these individuals in order to explore whether a history of one psychiatric condition might predispose individuals to further mental health disorders, especially in the context of added stress and strife in their life with a cancer diagnosis. Therefore, the findings regarding depression and anxiety prevalence and associated factors should be interpreted with consideration for this specific population subgroup. In future studies, we recognise that it may be prudent to include the type of pre-cancer mental health diagnosis which could help mitigate the confounding effects on results.

## Conclusion

Our study demonstrated that patients with cancer exhibited significant evidence of depression and anxiety and showed an increased rate of suicidality especially among patients with previous psychiatric histories. This demonstrates the importance of screening all patients with cancer for depression and anxiety. It also sheds light on a high-risk category of patients that requires individualised interventions and psychological support programs tailored to their physical and psychological well-being since diagnosis. This study also highlights the importance of having psycho-oncology programs in cancer centres to risk stratify patients at initial diagnosis and to integrate screening and treatment of mental disorders as part of the treatment protocols for patients with cancer.

## Conflicts of interest

The authors declare that they have no conflicts of interest.

## Institutional review

This study was approved by the Institutional Review Board committee of the AUBMC (protocol ID: SBS-2020-0273).

## Funding

No funding was received while preparing this work.

## Author contributions

Conceptualisation: MAH, AEM and HA

Manuscript draft: MAH, AEM and MB.

Data collection and analysis: AEM, SK, TK and NB.

Manuscript revision: RAZ and GA.

All authors have read and approved the final manuscript and contributed to the article and approved the submitted version.

## Figures and Tables

**Table 1.** Patient baseline characteristics at diagnosis.

**Total number of patients**	194	
Age	X̄: 55.7 (±15.69)	Range <18–90>
Gender		
43.9% male patients	56.1% female patients	
Type of malignancy		
Breast cancer	21.6%	
Gastrointestinal cancer	25.3%	
Lung cancer	12.9%	
Hematologic	22.7%	
Other: less common tumours like sarcoma, melanoma, skin tumours and nasopharyngeal carcinoma etc.	17.5%	
Disease status at diagnosis		
Loco-regional	63%	
Advanced	37%	
ECOG performance status		
0–1	91.8%	
2	8.2%	
Treatment intent		
Curative intent	93%	
Palliative intent	7%	
Treatment modalities		
Chemotherapy	88.1%	
Radiotherapy	22.1%	
Immunotherapy	7.2%	
Hormonal therapy	3.1%	
Targeted therapy	19.1%	
Required surgery during disease course	31.4%	
Treatment route		
Intravenous	85.6%	
Oral	2.1%	
Both	6.7%	
Number of hospitalisations		
0	56.2%	
1	28.9%	
2 or more	14.9%	
Psychiatric history		
Patients had personal history of mental health problems (including major depressive disorder) prior to being diagnosed with cancer	16%	
Sought a professional mental health provider (psychiatrist, psychologist, therapist, counsellor, or life coach) prior to diagnosis	17%	
Taking a psychiatric medication (including antidepressants) that was started prior to being diagnosed with cancer	13.4%	

**Table 2.** The prevalence of depression and anxiety among the population.

Anxiety measured by GAD-7	
No anxiety	48.5%
Minimum level of anxiety	36.6%
Moderate or severe levels of anxiety (score of more than 10)	14.9%
Depression measured by PHQ-9	
No depression	36.6%
Mild depression	40.7%
Moderate to severe depression	22.7%
Suicidality: patient clearly stated wishes for death	
Never had suicidal thoughts	93.8%
Had suicidal thoughts	6.2%

**Table 3.** Multivariate analysis for risk factors for development of depression, anxiety and suicidality.

**Depression**				
History of seeking help for mental health	*B* = 1.381	SE = 0.440	*p* = 0.002	CI = 1.680–9.415
Complications	*B* = 1.111	SE = 0.479	*p* = 0.20	CI = 1.187–7.777
Radiotherapy	*B* = 0.962	SE = 0.433	*p* = 0.025	CI = 1.120–6.118
ECOG	*B* = 1.669	SE = 0.617	*p* = 0.007	CI = 1.582–17.797
**Anxiety**				
History of seeking help for mental health	*B* = 1.176	SE = 0.456	*p* = 0.01	CI = 1.086–9.255
**Suicidality**				
History of seeking help for mental health	*B* = 2.128	SE = 0.623	*p* = 0.001	CI = 2.479–28.465

## References

[1] Grunfeld E, Coyle D, Whelan T (2004). Family caregiver burden: results of a longitudinal study of breast cancer patients and their principal caregivers. CMAJ.

[2] Mitchell AJ, Chan M, Bhatti H (2011). Prevalence of depression, anxiety, and adjustment disorder in oncological, haematological, and palliative-care settings: a meta-analysis of 94 interview-based studies. Lancet Oncol.

[3] Naser AY, Hameed AN, Mustafa N (2021). Depression and anxiety in patients with cancer: a cross-sectional study. Front Psychol.

[4] Research WHOIAfC (2020). Lebanon GLOBOCAN 2020.

[5] Andersen BL, DeRubeis RJ, Berman BS (2014). Screening, assessment, and care of anxiety and depressive symptoms in adults with cancer: an American Society of Clinical Oncology guideline adaptation. J Clin Oncol.

[6] Burgess C, Cornelius V, Love S (2005). Depression and anxiety in women with early breast cancer: five year observational cohort study. BMJ.

[7] Civilotti C, Acquadro Maran D, Santagata F (2020). The use of the distress thermometer and the hospital anxiety and depression scale for screening of anxiety and depression in Italian women newly diagnosed with breast cancer. Support Care Cancer.

[8] Karam EG, Mneimneh ZN, Dimassi H (2008). Lifetime prevalence of mental disorders in Lebanon: first onset, treatment, and exposure to war. PLoS Med.

[9] Baskar R, Lee KA, Yeo R (2012). Cancer and radiation therapy: current advances and future directions. Int J Med Sci.

[10] Wagner LI, Pugh SL, Small Jr W (2017). Screening for depression in cancer patients receiving radiotherapy: feasibility and identification of effective tools in the NRG Oncology RTOG 0841 trial. Cancer.

[11] Stiegelis HE, Ranchor AV, Sanderman R (2004). Psychological functioning in cancer patients treated with radiotherapy. Patient Educ Couns.

[12] Kroenke K, Spitzer RL, Williams JB (2001). The PHQ-9: validity of a brief depression severity measure. J Gen Intern Med.

[13] Spitzer RL, Kroenke K, Williams JB (2006). A brief measure for assessing generalized anxiety disorder: the GAD-7. Arch Intern Med.

